# Magnetic Structure and Propagation of Two Interacting CMEs From the Sun to Saturn

**DOI:** 10.1029/2021JA029770

**Published:** 2021-11-03

**Authors:** Erika Palmerio, Teresa Nieves‐Chinchilla, Emilia K. J. Kilpua, David Barnes, Andrei N. Zhukov, Lan K. Jian, Olivier Witasse, Gabrielle Provan, Chihiro Tao, Laurent Lamy, Thomas J. Bradley, M. Leila Mays, Christian Möstl, Elias Roussos, Yoshifumi Futaana, Adam Masters, Beatriz Sánchez‐Cano

**Affiliations:** ^1^ Space Sciences Laboratory University of California–Berkeley Berkeley CA USA; ^2^ CPAESS University Corporation for Atmospheric Research Boulder CO USA; ^3^ Heliophysics Science Division NASA Goddard Space Flight Center Greenbelt MD USA; ^4^ Department of Physics University of Helsinki Helsinki Finland; ^5^ STFC RAL Space Rutherford Appleton Laboratory Harwell Campus Oxfordshire UK; ^6^ Solar–Terrestrial Centre of Excellence—SIDC Royal Observatory of Belgium Brussels Belgium; ^7^ Skobeltsyn Institute of Nuclear Physics Moscow State University Moscow Russia; ^8^ ESTEC European Space Agency Noordwijk The Netherlands; ^9^ School of Physics and Astronomy University of Leicester Leicester UK; ^10^ National Institute of Information and Communications Technology (NICT) Koganei Japan; ^11^ LESIA Observatoire de Paris PSL CNRS UPMC Université Paris Diderot Meudon France; ^12^ LAM Pythéas Aix Marseille Université CNRS CNES Marseille France; ^13^ Space Research Institute Austrian Academy of Sciences Graz Austria; ^14^ Institute of Geodesy Graz University of Technology Graz Austria; ^15^ Max Planck Institute for Solar System Research Göttingen Germany; ^16^ Swedish Institute of Space Physics Kiruna Sweden; ^17^ The Blackett Laboratory Imperial College London London UK

**Keywords:** coronal mass ejections, heliophysics, solar wind, interplanetary magnetic field, heliosphere

## Abstract

One of the grand challenges in heliophysics is the characterization of coronal mass ejection (CME) magnetic structure and evolution from eruption at the Sun through heliospheric propagation. At present, the main difficulties are related to the lack of direct measurements of the coronal magnetic fields and the lack of 3D in‐situ measurements of the CME body in interplanetary space. Nevertheless, the evolution of a CME magnetic structure can be followed using a combination of multi‐point remote‐sensing observations and multi‐spacecraft in‐situ measurements as well as modeling. Accordingly, we present in this work the analysis of two CMEs that erupted from the Sun on April 28, 2012. We follow their eruption and early evolution using remote‐sensing data, finding indications of CME–CME interaction, and then analyze their interplanetary counterpart(s) using in‐situ measurements at Venus, Earth, and Saturn. We observe a seemingly single flux rope at all locations, but find possible signatures of interaction at Earth, where high‐cadence plasma data are available. Reconstructions of the in‐situ flux ropes provide almost identical results at Venus and Earth but show greater discrepancies at Saturn, suggesting that the CME was highly distorted and/or that further interaction with nearby solar wind structures took place before 10 AU. This work highlights the difficulties in connecting structures from the Sun to the outer heliosphere and demonstrates the importance of multi‐spacecraft studies to achieve a deeper understanding of the magnetic configuration of CMEs.

## Key Points

- We analyze the eruption of two coronal mass ejections on April 28, 2012 and their evolution up to Saturn
- We study and compare the flux rope magnetic structure at the Sun, at Venus, at Earth, and at Saturn
- We find a single flux rope structure at all planets, suggesting interaction of the two eruptions in the inner heliosphere

## 1 Introduction

Coronal mass ejections (CMEs; e.g., Webb & Howard, 2012) are spectacular eruptions of magnetic fields and plasma that are regularly launched from the Sun throughout the heliosphere. Their magnetic structure, when they leave the solar atmosphere, is that of a flux rope (e.g., Chen, 2011; Forbes, 2000; Green et al., 2018; Klimchuk, 2001), that is consisting of a bundle of magnetic fields wrapped about a central axis. After erupting, CMEs usually undergo rapid acceleration and expansion in the low corona (e.g., Patsourakos, Vourlidas, & Kliem, 2010; Patsourakos, Vourlidas, & Stenborg, 2010; Temmer et al., 2008, 2010; Veronig et al., 2018) as a result of the large energy release and the high internal pressure compared to that of the ambient solar wind (Démoulin & Dasso, 2009). After the initial, impulsive phase, that is from the outer corona outwards, CMEs generally expand in a self‐similar fashion (e.g., Good et al., 2019; Schwenn et al., 2005; Subramanian et al., 2014; Vršnak et al., 2019). The speed at which CMEs propagate depends on the speed of the surrounding solar wind flow, which has the effect of accelerating slower CMEs and decelerating faster CMEs (e.g., Gopalswamy et al., 2000; Vršnak & Žic, 2007). CME expansion in interplanetary space is believed to usually take place up to ∼10–15 AU, where CMEs reach pressure balance with the solar wind (e.g., J. D. Richardson et al., 2006; von Steiger & Richardson, 2006). As CMEs propagate through the outer heliosphere, they may interact with stream interaction regions (SIRs; e.g., I. G. Richardson, 2018) or with other CMEs to produce merged interaction regions (MIRs; e.g., Burlaga et al., 1986, 1997), which are believed to dominate the structure of the heliosphere at large heliocentric distances (e.g., Gazis et al., 2006; von Steiger & Richardson, 2006).

In reality, the sparsity of observations throughout the heliosphere, together with the fact that in‐situ measurements typically sample a 1D trajectory through a much larger structure, mean that many aspects of CME evolution are yet to be fully understood (for recent reviews on CME evolution, see Luhmann et al., 2020; Manchester et al., 2017). For example, it is unclear to which extent 1D in‐situ measurements are representative of the global CME structure (e.g., Al‐Haddad et al., 2011; Owens et al., 2017), mainly because of distortions (e.g., Manchester et al., 2004; Owens, 2008; Savani et al., 2010) and/or the particular sampling distance with respect to the CME nose and central axis (e.g., Cane et al., 1997; Kilpua et al., 2011; Marubashi & Lepping, 2007). As they propagate through the solar corona and interplanetary space, CMEs are also known to experience deflections and rotations (e.g., Isavnin et al., 2014; Kay et al., 2015; Vourlidas et al., 2011; Y. Wang et al., 2004), which may significantly affect the magnetic configuration that is later measured in situ (e.g., Palmerio et al., 2018; Yurchyshyn, 2008). Furthermore, in addition to the difficulties in understanding CME evolution for single‐CME events, cases where CMEs interact with solar wind structures (e.g., Heinemann et al., 2019; Rouillard et al., 2010; Winslow et al., 2016, 2021) or with other CMEs (e.g., Dasso et al., 2009; Farrugia & Berdichevsky, 2004; Lugaz, Temmer, et al., 2017; Scolini et al., 2020) are not infrequent, thus complicating things further.

The properties of interplanetary CMEs (or ICMEs; e.g., Kilpua et al., 2017) have been studied mainly around 1 AU (e.g., Cane & Richardson, 2003; Jian et al., 2006, 2018; Nieves‐Chinchilla, Vourlidas, et al., 2018; Nieves‐Chinchilla et al., 2019; Owens, 2018; Regnault et al., 2020; I. G. Richardson & Cane, 2010), that is where continuous in‐situ measurements of the solar wind have been available for several decades and up to this day. In particular, the subset of ICME ejecta known as magnetic clouds (Burlaga et al., 1981) have been analyzed extensively around Earth's orbit, both in statistical (e.g., Huttunen et al., 2005; Janvier, Démoulin, & Dasso, 2014; Li et al., 2014, 2018; Lynch et al., 2003; Nieves‐Chinchilla & Viñas, 2008; Nieves‐Chinchilla et al., 2005; Rodriguez et al., 2016; Wood et al., 2017) and in case studies (e.g., Kilpua et al., 2009; Lynch et al., 2010; Möstl et al., 2008; Möstl, Farrugia, Miklenic, et al., 2009; Nieves‐Chinchilla et al., 2011). Magnetic clouds are characterized by enhanced magnetic field magnitudes, a large and smooth rotation of the magnetic field over one direction, low proton temperatures, and low plasma beta, and they are of particular interest because their magnetic configuration corresponds to that of a flux rope. Interestingly, the first study that identified and defined magnetic clouds in the solar wind, that is that of Burlaga et al. (1981), was performed using observations from several spacecraft between 1 and 2 AU, thus highlighting the importance of well‐separated multi‐spacecraft measurements to understand the intrinsic structure of CMEs in the heliosphere.

Indeed, studying the properties of CMEs at different radial distances as they propagate throughout the heliosphere can provide paramount information and insight into CME evolution. Heliocentric orbiters such as Helios 1/2 and Ulysses have enabled long‐term observation and analysis of ICMEs away from 1 AU and/or the ecliptic plane (e.g., Bothmer & Schwenn, 1994, 1998; Du et al., 2010; Jian et al., 2008b; Lepri & Zurbuchen, 2004; Y. Liu et al., 2005; I. G. Richardson, 2014; Riley et al., 2000). Data from the more recently launched Parker Solar Probe, BepiColombo, and Solar Orbiter have resulted in several studies of CMEs measured between the Sun and 1 AU (e.g., E. E. Davies et al., 2021; Korreck et al., 2020; Lario et al., 2020; Nieves‐Chinchilla et al., 2020; Zhao et al., 2020; Weiss et al., 2021). Considerable results were also achieved using data from spacecraft, such as Pioneer 10/11 and Voyager 1/2, that were launched on escape trajectories out of the solar system and that have provided valuable information on the behavior of ICMEs at progressively larger heliocentric distances (e.g., Burlaga et al., 2001; C. Wang & Richardson, 2001, 2004). Finally, another important contribution in understanding CME properties away from 1 AU has come from planetary missions, which usually spend some part of their orbit outside of the planet's bow shock, thus exposing their instruments to the solar wind (e.g., Collinson et al., 2015; Good & Forsyth, 2016; Janvier et al., 2019; Jian et al., 2008a; Lee et al., 2017; Palmerio et al., 2021; Winslow et al., 2015).

In‐situ measurements of the same CMEs by multiple spacecraft at different radial distances throughout the heliosphere enable studies of their interplanetary evolution (e.g., Burlaga et al., 1981; E. E. Davies et al., 2020; de Lucas et al., 2011; Good et al., 2018, 2019; Lugaz et al., 2020; Salman et al., 2020; Vršnak et al., 2019). Additionally, solar and heliospheric remote‐sensing observations can be combined with multi‐spacecraft in‐situ data to obtain a complete picture of the whole solar–heliospheric system when characterizing CME evolution (e.g., Asvestari et al., 2021; Kilpua et al., 2019; Möstl et al., 2015; Nieves‐Chinchilla et al., 2012; Palmerio et al., 2021; Prise et al., 2015; J. D. Richardson et al., 2002; Rodriguez et al., 2008; Rouillard et al., 2009). In particular, the use of heliospheric imagery has been proven useful to connect CMEs at the Sun to their in‐situ counterparts (e.g., DeForest et al., 2013; Möstl, Farrugia, Temmer, et al., 2009; Möstl et al., 2017; Palmerio et al., 2019; Rouillard, 2011; Srivastava et al., 2018). ICME structures can be identified in situ using a number of signatures based on magnetic field, plasma, compositional, and energetic particle data (e.g., Zurbuchen & Richardson, 2006). Furthermore, since spacecraft may lack magnetic field and/or plasma instruments (especially those not dedicated to studying the solar wind), reduction in galactic cosmic rays (GCR) measurements known as Forbush decreases (Forbush, 1937; Hess & Demmelmair, 1937) can be used as a proxy for ICME passage (e.g., Cane, 2000; Dumbović et al., 2020; Freiherr von Forstner et al., 2018, 2020; Papaioannou et al., 2020; I. G. Richardson & Cane, 2011; Winslow et al., 2018; Witasse et al., 2017).

Despite the amount of spacecraft scattered throughout the heliosphere and decades of research efforts, the number of well‐observed multi‐spacecraft events covering extended radial distances into the outer heliosphere is still exiguous, even when considering both studies of the solar wind and interplanetary magnetic field (e.g., Burlaga et al., 2001; Prise et al., 2015; Witasse et al., 2017) as well as multi‐planet observing campaigns designed to track the auroral response of giant planetary magnetospheres to CME‐driven shocks between 5 and 30 AU (e.g., Branduardi‐Raymont et al., 2013; Dunn et al., 2021; Lamy et al., 2012, 2017; Lamy, 2020; Prangé et al., 2004). More so, even fewer studies have focused on the magnetic structure of CMEs in the outer heliosphere. The Bastille event studied by Burlaga et al. (2001) at Voyager 2 at 63 AU is considered the most distant magnetic cloud ever observed, but its handedness was reported to be opposite to that of the corresponding ejecta at 1 AU, thus highlighting the difficulties in associating measurements that are separated by large radial distances.

**Figure 1:**
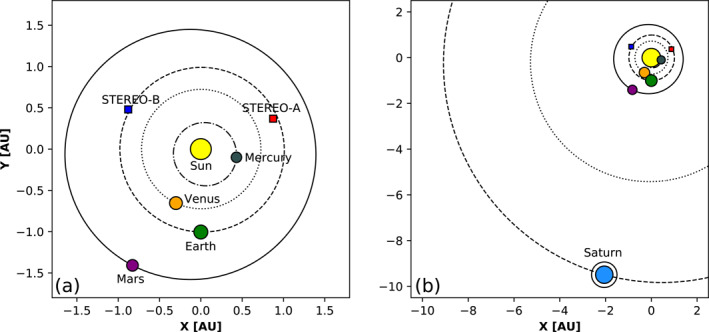
Position of various planets and spacecraft until the orbits of (a) Mars and (b) Saturn, on April 28, 2012. The orbits of all planets from Mercury to Saturn are also indicated.

This work represents the first in‐depth study of the magnetic structure of a (merged) flux rope from the Sun to ∼10 AU. We analyze here in detail the eruption and evolution of two CMEs that left the Sun on 28 April 2012 and were observed to interact in the inner heliosphere. The favorable position of the spacecraft involved in this study allows us to follow the event in remote‐sensing imagery as it propagates away from the Sun and then to study its in‐situ signatures and magnetic structure at Venus, Earth, and Saturn. Figure 1 shows the position of various planets and spacecraft throughout the heliosphere on the day of the eruptions under analysis. This article is organized as follows. In Section 2, we enumerate the various instruments that are involved in this study. In Section 3, we describe the CMEs under analysis from a remote‐sensing observational perspective. In Section 4, we estimate the propagation of the CMEs and their impact at different locations using the remote‐sensing observations described in Section 3 as input. In Section 5, we present the in‐situ signatures and analyze the magnetic structure of the interacting CMEs at different heliocentric distances. In Section 6, we discuss different aspects of the event, especially in terms of CME propagation and magnetic structure. Finally, in Section 7, we summarize our results and present our conclusions.

## 2 Spacecraft and Ground‐Based Data

We list here the fleet of instruments that are involved in this study, starting from the Sun and moving outwards to Saturn. We combine remote‐sensing and in‐situ data in order to follow and characterize the event at various locations throughout the heliosphere.

Observations of the solar disc are made from three vantage points, namely Earth and the twin Solar Terrestrial Relations Observatory (STEREO; Kaiser et al., 2008) spacecraft. The STEREO mission comprises two identical spacecraft that orbit the Sun close to 1 AU, one ahead of Earth in its orbit (STEREO‐A) and the other one trailing behind (STEREO‐B, which has been out of contact since late 2014). At the time of the events under analysis, STEREO‐A and ‐B were located ∼113° west and ∼118° east of the Sun–Earth line, respectively. Extreme ultra‐violet (EUV) images are provided by the Atmospheric Imaging Assembly (AIA; Lemen et al., 2012) onboard the Solar Dynamics Observatory (SDO; Pesnell et al., 2012) orbiting Earth and the Sun Earth Connection Coronal and Heliospheric Investigation (SECCHI; R. A. Howard et al., 2008) Extreme UltraViolet Imager (EUVI) onboard STEREO. Line‐of‐sight magnetograms are available from Earth's viewpoint only, and we use data from the Helioseismic and Magnetic Imager (HMI; Scherrer et al., 2012) onboard SDO.

Coronagraph observations are made from the same three viewpoints. White‐light images from Earth are provided by the Large Angle and Spectrometric Coronagraph (LASCO; Brueckner et al., 1995) C2 (2.2–6 $R_{⊙}$) and C3 (3.5–30 $R_{⊙}$) cameras onboard the Solar and Heliospheric Observatory (SOHO: Domingo et al., 1995). Imagery from STEREO‐A and ‐B is supplied by the SECCHI/COR1 (1.5–4 $R_{⊙}$) and COR2 (2.5–15 $R_{⊙}$) coronagraphs.

White‐light observations of the heliosphere are provided by the Heliospheric Imagers (HI; Eyles et al., 2009) onboard the twin STEREO spacecraft. Each HI instrument consists of two cameras, HI1 (4–24°) and HI2 (18–88°), that image interplanetary space in the vicinity of the Sun–Earth line (the degrees indicate the elongation in helioprojective radial coordinates).

In‐situ measurements from Venus are provided by the Venus Express (VEX; Svedhem et al., 2007) spacecraft. The instruments that we avail ourselves of are the Magnetometer (MAG; Zhang et al., 2006) and the Analyzer of Space Plasmas and Energetic Atoms (ASPERA‐4; Barabash et al., 2007). From the ASPERA‐4 package, we use data taken by the Ion Mass Analyzer (IMA) and the Electron Spectrometer (ELS) sensors. VEX terminated operations in early 2015 and was characterized by a 24‐h highly elliptical orbit, most of which was in the solar wind and the remaining couple of hours were spent inside the bow shock of Venus. MAG was operational at all times, whilst ASPERA‐4 was turned on for several hours close to periapsis and apoapsis.

In‐situ measurements from Earth are provided by the Wind (Ogilvie & Desch, 1997) spacecraft, which orbits the Sun from Earth's Lagrange L1 point. Magnetic field and plasma (including solar wind electron distributions) data are taken by the Magnetic Fields Investigation (MFI; Lepping et al., 1995) and Solar Wind Experiment (SWE; Ogilvie et al., 1995) instruments. We also use count rate data from the Neutron Monitor Database (NMDB), and specifically from the Thule (THUL), Oulu (OULU), and South Pole (SOPO) observatories on ground, as GCR proxies.

Finally, in‐situ measurements from Saturn are provided by the Cassini (Matson et al., 2002) spacecraft. We use data from the Cassini Magnetic Field Investigation (MAG; Dougherty et al., 2004), the Radio and Plasma Wave Science (RPWS; Gurnett et al., 2004) experiment, and the Magnetosphere Imaging Instrument (MIMI; Krimigis et al., 2004). MIMI consists of three different sensors, and we use data from the Low Energy Magnetospheric Measurement System (LEMMS) sensor. The Cassini spacecraft was dismissed in 2017 through a controlled entry into Saturn.

## 3 Remote‐Sensing Observations

In this section, we provide an overview of the solar events that originated on April 28, 2012 and follow them from the solar disc (Section 3.1) through white‐light imagery of the corona (Section 3.2) and heliosphere (Section 3.3).

### 3.1 Solar Disc Observations

**Figure 2:**
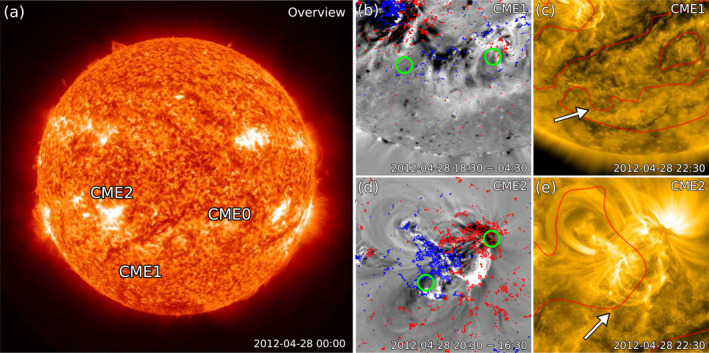
Eruptive events on April 28, 2012 as seen in solar disc imagery from SDO. (a) Overview of the solar disc in the 304 Å channel prior to the eruptions, with the approximate source regions of the three CMEs marked in chronological order as “CME0,” “CME1,” and “CME2,” respectively. (b) Base‐difference image in the 211 Å channel overlaid with magnetogram contours (red: positive polarity, blue: negative polarity). The footpoints of CME1 are indicated with green circles. (c) Image in the 171 Å channel showing the post‐eruption arcade signatures of CME1. The global neutral lines (calculated from smoothed magnetogram data taken at the same time) are overlaid in red, and the main polarity inversion line involved in the eruption is indicated with a white arrow. (d–e) Same as panels (b–c), but for CME2.

The sequence of events analyzed in this article commenced on April 28, 2012, which was a day characterized by several eruptions. Here, we focus on the three major solar events that originated on that day from the Earth‐facing disc. Figure 2a shows the approximate source regions of these CMEs as seen by SDO (i.e., from Earth's perspective). For a complete set of observations of the front‐sided eruptions of April 28, 2012 from three viewpoints (STEREO‐A, Earth, and STEREO‐B), see Movie S1.

The first major eruption of the day, marked as “CME0” in Figure 2a, had its onset at ∼10:00 UT and originated from a quiet‐Sun, J‐shaped filament located in the southwestern quadrant of the solar disc (approximately at S25W40). Given the large scale of the resulting eruption and the proximity of the filament to the source of the next CME (CME1), it is possible that CME0 triggered the chain of subsequent events (e.g., Schrijver & Title, 2011; Török et al., 2011). However, this CME was seen to deflect significantly toward the southwest already in the lower corona (see Movie S1 and Section 3.2), making it highly unlikely to interact with the following CMEs or to encounter any observer close to the ecliptic plane. Hence, CME0 will be disregarded for the rest of this study.

The following eruption, marked as “CME1” in Figure 2a, had its onset at ∼13:00 UT and originated from a quiet‐Sun, U‐shaped filament located in the southeastern quadrant of the solar disc (approximately at S45E15). Although this CME erupted from higher latitudes than CME0, it deflected toward the equator (see Movie S1 and Section 3.2), making it more likely to exhibit a significant component along the ecliptic plane and to interact with the following CME2. Hence, we proceed to evaluate the magnetic structure of the flux rope associated with CME1, also known as the “intrinsic flux rope type.” This can be achieved via a combination of multi‐wavelength, remote‐sensing observations that yield information on the chirality (or handedness), tilt, and axial field direction of a flux rope during eruption (see Palmerio et al., 2017, and references therein for a summary of the available proxies). The eruption of CME1 was not characterized by a clear pair of coronal dimmings (usually tracers of a flux rope's footpoints; e.g., B. J. Thompson et al., 2000; Zhukov & Auchère, 2004), but rather by several patches of diffuse dimming regions. Hence, we estimate the CME footpoints to be simply located at either end of the erupting filament's spine, noting however that these are approximate locations because of possible projection effects. The resulting footpoints are shown in Figure 2b, according to which CME1 was rooted in a positive (negative) polarity to the west (east). Furthermore, we observe the “roll effect” (i.e., the sense of bending and twisting) of the filament material off limb in STEREO imagery (see Movie S1), which can be used as a chirality proxy (e.g., Martin, 2003; Panasenco & Martin, 2008). We determine that the filament rolled in a right‐handed sense, which is also confirmed by the presence of a left‐bearing barb (signature of a right‐handed CME; e.g., Martin, 1998) that we could identify in Hα imagery of the solar disc from the Big Bear Solar Observatory (not shown). For the the flux rope tilt, we consider the inclination of the corresponding polarity inversion line and post‐eruption arcade (e.g., Marubashi et al., 2015), shown in Figure 2c. Both are inclined ∼15° counterclockwise with respect to the solar equator, implying thus a low‐inclination flux rope. A right‐handed, low‐inclination flux rope with an eastward axial field yields a north–east–south (NES) type, following the scheme of Bothmer and Schwenn (1998) and Mulligan et al. (1998).

Finally, the last eruption, marked as “CME2” in Figure 2a, had its onset at ∼18:00 UT and originated from active region (AR) 11469 (approximately at S15E20), in the vicinity of the eastern footpoint of the CME1 filament. Given the close location of the source region with respect to the center of the solar disc and noting no major deflections during early evolution (see Movie S1 and Section 3.2), we can expect CME2 to be likely Earth‐directed. Hence, we again estimate the intrinsic flux rope type associated with this eruption. Low‐coronal signatures of the CME included J‐shaped ribbons (shown in Figure 2d), proxies of right‐handed chirality (e.g., Démoulin et al., 1996). Together with coronal dimming pairs, flare ribbons are also tracers of where a flux rope is rooted (e.g., Aulanier et al., 2012; Janvier, Aulanier, et al., 2014), resulting in the footpoints indicated in Figure 2d and showing the northern (southern) leg anchored to the positive (negative) magnetic polarity. The polarity inversion line and post‐eruption arcade are both inclined ∼55° counterclockwise with respect to the solar equator, indicating a high‐to‐intermediate inclination flux rope. A right‐handed, high‐inclination flux rope with a southward axial field yields an east–south–west (ESW) type, whilst its low‐inclination counterpart would be a NES type.

### 3.2 Coronagraph Observations

**Figure 3:**
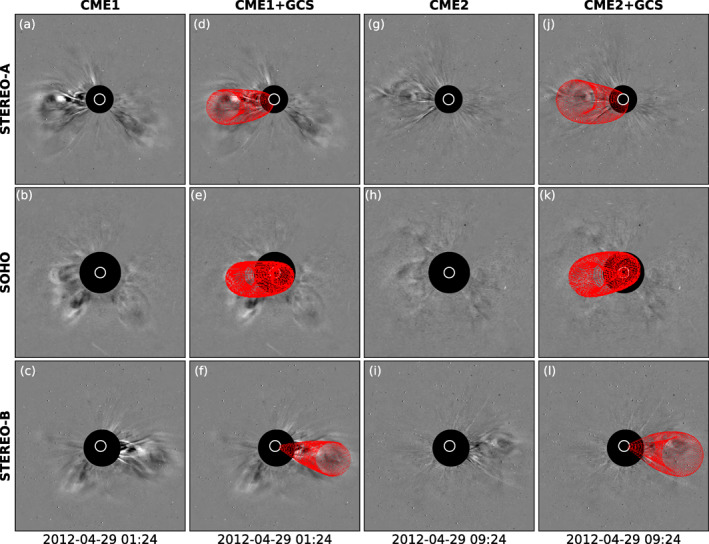
Coronagraph observations and GCS reconstructions for CME1 and CME2. (a–c) CME1 shown in difference images from the COR2‐A, C3, and COR2‐B telescopes, respectively. Background images are taken 1 h prior to each of the main images. (d–f) Same images as (a–c), with the GCS wireframe overlaid in red. (g–i) Same as (a–c), but for CME2. (j–l) Same as (d–f), but for CME2. All images are taken within 6 min of the times reported under each column.

After erupting, the April 28, 2012 CMEs appeared in coronagraph imagery from three locations (STEREO‐A, SOHO, and STEREO‐B). We provide here an overview of white‐light observations of the events throughout the solar corona, with a particular focus on the derivation of geometric and kinematic parameters for the eruptions that we deemed in Section 3.1 to be possibly Earth‐directed (i.e., CME1 and CME2, shown in Figure 3). For a complete set of the coronagraph observations from the three available viewpoints, see Movie S2.

As mentioned in Section 3.1, CME0 (the first to erupt and visible in COR2‐A imagery starting at 12:24 UT on April 28, 2012) was seen to propagate mainly below the ecliptic plane (STEREO views) and toward the west (SOHO view), strongly suggesting that the eruption was directed away from the Sun–Earth line and from the following CMEs. This event was particularly faint in STEREO‐B imagery, which is not surprising since the CME was propagating fully away with respect to the plane of the sky.

CME1 emerged in coronagraph imagery a few hours after CME0 (around 18:24 UT in COR2‐A data) and was seen to propagate at significantly lower latitudes than the previous eruption (see also Section 3.1). In addition, the main “bulk” of CME1 was preceded by a smaller feature at its southern edge, resulting in a double‐lobe structure visible from all viewpoints shown in Movie S2. We note that asymmetric and complex white‐light morphologies of CMEs that originated from filament eruptions have been reported in previous studies (e.g., Palmerio et al., 2021; Yang et al., 2012; Zhu et al., 2014). In the case under study, it is likely that the double‐lobe structure seen in STEREO imagery resulted from an uneven eruption and disconnection of the corresponding filament legs (e.g., R. Liu et al., 2009; Tripathi et al., 2006; Vourlidas et al., 2011). Furthermore, we note that images of CME1 from the SOHO viewpoint (see e.g., Figure 3b) are particularly complex to interpret because of an almost‐simultaneous back‐sided CME (clearly visible in Figures 3a and 3c) overlapping due to projection effects. Overall, CME1 did not appear to evolve drastically throughout the solar corona, suggesting that its orientation was maintained similar to that prior to eruption (cf. the low‐inclination configuration that was inferred from the analysis of solar disc imagery in Section 3.1).

Finally, CME2 appeared in coronagraph imagery a few hours after CME1 (around 22:24 UT in COR2‐A data) and was seen to propagate mainly along the ecliptic plane. It was the faintest of the three eruptions in white‐light data from all available viewpoints (see Movie S2), possibly because of its passage through an already disturbed corona due to the preceding CME1. The morphology of the eruption in coronagraph images from the two STEREO viewpoints (see also Figures 3g–3i) is reminiscent of a low‐inclination flux rope seen edge on (see, e.g., T. A. Howard et al., 2017; Krall & St. Cyr, 2006; Thernisien et al., 2006), suggesting that the CME rotated slightly upon eruption (cf. the high‐to‐intermediate‐inclination configuration that was inferred from the analysis of solar disc imagery in Section 3.1).

In order to obtain quantitative estimates of the geometric and kinematic parameters of CME1 and CME2 through the solar corona, we reconstruct both eruptions using the Graduated Cylindrical Shell (GCS; Thernisien et al., 2009; Thernisien, 2011) model. In the GCS model, a parameterized shell reminiscent of a croissant with its legs attached to the Sun is manually fitted to nearly simultaneous white‐light images until its morphology best matches the observed features. GCS model results are shown in Figures 3d–3f for CME1 and Figure 3j–3l for CME2. According to our reconstruction results, CME1 had a propagation direction of (θ,ϕ) = (−12°,−18°) and a tilt (γ) of 5° with respect to the solar equatorial plane, whilst the corresponding values for CME2 are (θ,ϕ) = (−8°,−24°) and γ=15° (here, θ and ϕ are expressed in Stonyhurst coordinates; e.g., W. T. Thompson, 2006, and a positive γ is assumed for counterclockwise rotations). Furthermore, we determine the speeds (v) of both CMEs through the outer corona by performing GCS reconstructions at two separate times separated by 1 h, resulting in v=405.8 km$\cdot s^{-1}$ for CME1 and v=309.2 km$\cdot s^{-1}$ for CME2. Overall, both CMEs propagated slightly toward the southeast as seen from Earth, had low speeds, and were characterized by a low tilt of their axes with respect to the solar equatorial plane. In terms of their resulting magnetic configuration through the corona, it can be assumed that CME1 maintained its NES flux rope type determined in Section 3.1, whilst CME2 rotated from its higher‐inclination, ESW type inferred in Section 3.1. The shortest “path” from γ=55° to γ=5° corresponds to a clockwise motion of the CME axis, which is the expected sense of rotation for a right‐handed flux rope (e.g., Green et al., 2007; Lynch et al., 2009). Hence, these results indicate that both CME1 and CME2 featured a NES flux rope configuration when they left the fields of view of the coronagraphs employed in this study.

### 3.3 HI Observations

**Figure 4:**
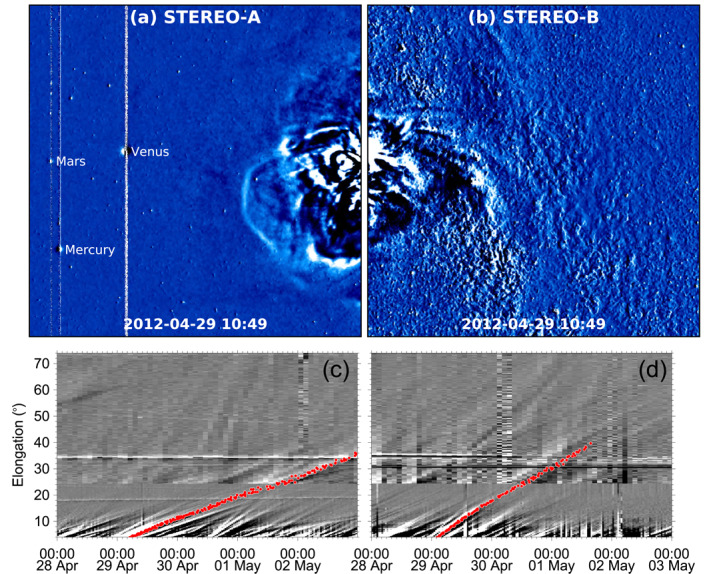
CME1 and CME2 seen as a single structure in images from the STEREO/SECCHI/HI cameras. (a–b) The CME seen in running‐difference images taken with the (a) HI1‐A and (b) HI1‐B cameras. The locations of Mercury, Venus, and Mars are marked in panel (a). (c–d) Time–elongation maps from (c) STEREO‐A and (d) STEREO‐B. The CME is tracked in red. The maps are constructed along position angles of 90° for STEREO‐A and 260° for STEREO‐B.

After leaving the field of view of the COR2 coronagraphs, CME1 and CME2 appeared in images from the HI cameras onboard both STEREO spacecraft. For a complete set of observations from both HI1 cameras, see Movie S3. An overview of HI observations is shown in Figure 4. CME1 first emerged in HI1‐A data at 20:49 UT on April 28, 2012 and in HI1‐B data at 22:49 UT on the same day. The complex morphology of CME1, mentioned already in Section 3.2, is evident in heliospheric imagery as well. Furthermore, it is difficult to clearly distinguish the front of CME2 amongst the material of the preceding CME1, making the two eruptions appear as a merged, complex structure as they propagate away from the Sun. Figures 4a and 4b shows a snapshot of the complex structure observed in HI1 imagery, whilst Figures 4c and 4d shows time–elongation maps constructed using data from both HI1 and HI2 cameras, together with the corresponding tracks of the merged CME.

We note that CME1 and CME2 are also indicated as a single CME in the HELiospheric Cataloging, Analysis and Techniques Service (HELCATS) catalogs, based on observations made with the STEREO/SECCHI/HI cameras. This event is included in the HICAT catalog (Harrison et al., 2018), which was compiled through visual inspection of HI1 images, and in the HIGeoCAT catalog (Barnes et al., 2019), which was compiled using time–elongation maps and by applying single‐spacecraft fitting techniques to derive CME kinematic properties. In both catalogs, CMEs are identified using single‐spacecraft data, hence the STEREO‐A and STEREO‐B observations are presented separately. Amongst the fitting techniques reported in HIGeoCAT, we consider here the results obtained with the Self‐Similar Expansion fitting technique (SSEF; J. A. Davies et al., 2012; Möstl & Davies, 2013) with a fixed half‐width of 30° applied to time–elongation single‐spacecraft data. In the SSEF model, CMEs are assumed to have a circular front and to propagate radially with constant speed and half‐width. SSEF results based on STEREO‐A observations report a propagation direction of (θ,ϕ) = (−16°,−9°) and a speed of 405 km$\cdot s^{-1}$; whilst SSEF results based on STEREO‐B observations report a propagation direction of (θ,ϕ) = (−16°,1°) and a speed of 791 km$\cdot s^{-1}$. Whilst the values for propagation latitude and longitude are consistent with each other and also with the GCS results shown in Section 3.2, the SSEF‐B speed is approximately twice as large as the SSEF‐A one, which is on the other hand basically identical to the speed for CME1 derived using GCS reconstructions.

## 4 CME Propagation Modeling

In this section, we estimate the impact locations and arrival times of CME1 and CME2 across the heliosphere. In order to achieve this, we employ two different propagation models that are based on different physical assumptions and observational inputs. The first model (presented in Section 4.1) is based uniquely on HI observations (see also Section 3.3) and treats CME1 and CME2 as a single, merged structure. In the second model (presented in Section 4.2) CME1 and CME2 are inserted separately and their input parameters are derived from coronagraph observations (see also Section 3.2).

### 4.1 SSSE Model

**Figure 5:**
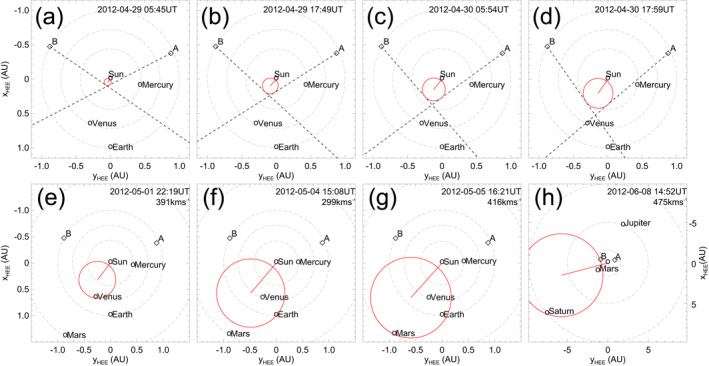
Schematic representation of the stereoscopic self‐similar expansion (SSSE) method applied to the merged CME1 and CME2 observed in HI data. (a–d) Position of the coronal mass ejection (CME) front (red circle) within the ecliptic plane triangulated from the observed leading edge in the HI1 cameras (dashed lines), shown at ∼12‐h intervals. (e–h) CME position extrapolated from the last observations to predict arrival times at four planets: (e) Venus, (f) Earth, (g) Mars, and (h) Saturn. Plots are shown in the Heliocentric Earth Ecliptic (HEE) coordinate system.

The first CME propagation model that we employ in this study is based on HI observations and fittings such as the SSEF results presented in Section 3.3. The SSEF results reported in the HELCATS catalogs, however, consider observations made with each spacecraft separately. Since the merged CME1 and CME2 were observed from both STEREO probes, we use here the two‐spacecraft version of the SSE model, that is the Stereoscopic Self‐Similar Expansion (SSSE; J. A. Davies et al., 2013) model. With the aid of time–elongation data (see Figure 4) from both spacecraft, we initially triangulate the CME front as it propagates away from the Sun assuming a circular cross‐section and fixed half‐width (set in this case as ω/2=60°, since the model was found by Barnes et al., 2020, to perform better with angular extents ≫30°). Then, in order to propagate the CME beyond its last observation time in both HI cameras, we fit a second‐order polynomial to the CME apex as a function of time, assuming a constant propagation direction. As a result, the merged CME is predicted to impact Venus (2012‐05‐01T22:19), Earth (2012‐05‐04T15:08), Mars (2012‐05‐05T16:21), and Saturn (2012‐06‐08T14:52). Figure 5 shows the position of the tracked CME front (top row) and the impact location at the four planets with respect to the apex (bottom row).

### 4.2 Enlil Simulation

**Figure 6:**
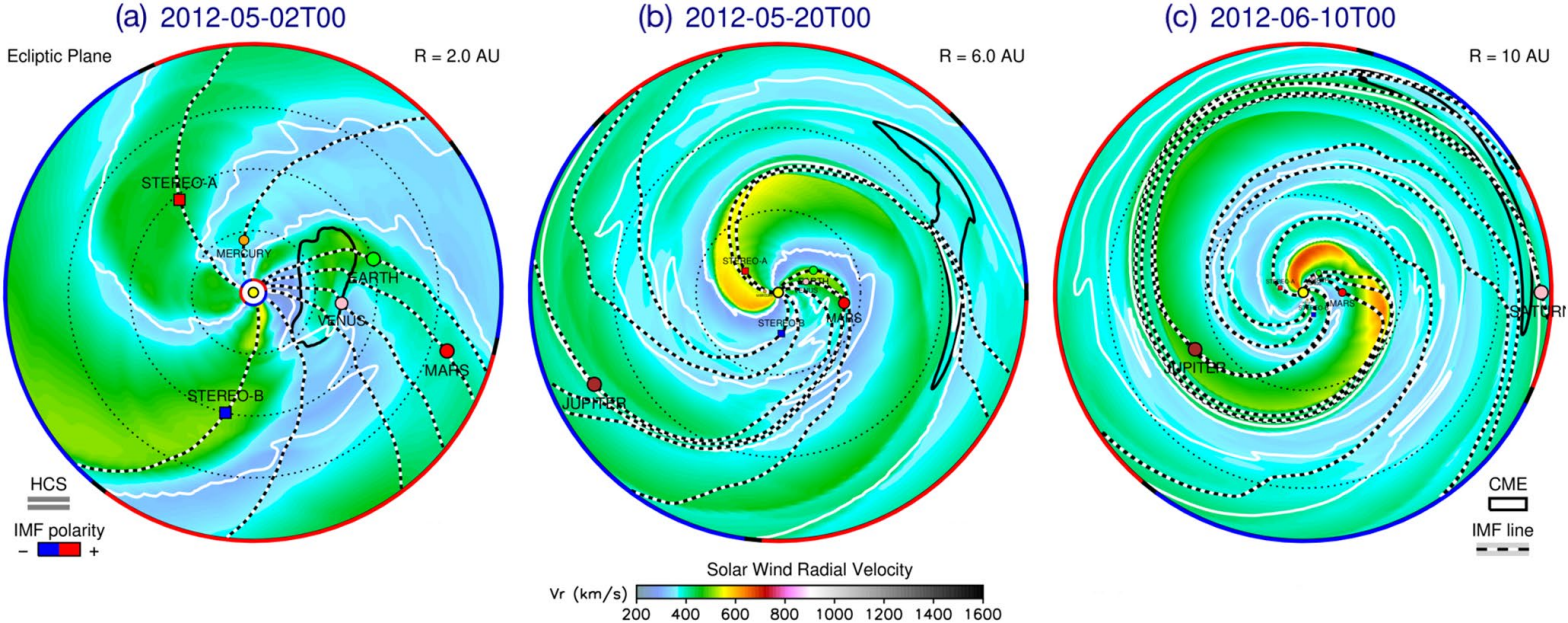
Screenshots from the WSA–Enlil + Cone simulation. The parameter shown in the plots is the solar wind radial speed in the ecliptic plane on (a) May 2, 2012, (b) May 20, 2012, and (c) June 10, 2012. The merged CME ejecta is represented with a black countour.

The second CME propagation model that we use in this work is the 3D heliospheric magnetohydrodynamic (MHD) Enlil (Odstrcil, 2003; Odstrcil et al., 2004) model. Enlil uses the Wang–Sheeley–Arge (WSA; Arge et al., 2004) coronal model to generate a background solar wind from its inner boundary (placed at 21.5 $R_{⊙}$ or 0.1 AU) onwards. Here, we set the outer boundary of the simulation domain at 10 AU. CMEs are launched through the heliospheric domain at the inner boundary as spherical hydrodynamic structures, that is lacking an internal magnetic field. The input parameters for CME1 and CME2 are entirely derived from the GCS reconstructions reported in Section 3.2. The two CMEs that we inject have an elliptical cross‐section and their angular extent is obtained by “cutting” a slice out of the GCS shell (see Figure 3 and Thernisien, 2011). The speeds and injection times are derived from the last observations in coronagraph data and by propagating the CMEs up to 21.5 $R_{⊙}$ under the assumption of constant speed. As a result, CME1 is launched on April 29, 2012 at 04:32 UT, at (θ,ϕ) = (−12°,−18°) and with axis tilt γ=5°, speed v=405.8 km$\cdot s^{-1}$, and half‐angular width ($R_{\max},R_{\min}$) = (41.6°,16.9°); CME2 is launched on April 29, 2012 at 12:42 UT, at (θ,ϕ) = (−8°,−24°) and with γ=15°, v=309.2 km$\cdot s^{-1}$, and ($R_{\max},R_{\min}$) = (44.8°,20.5°). Three screenshots from the simulation, corresponding to when the merged CME was at approximately 1, 5, and 10 AU, are shown in Figure 6. We note that, although CME2 is slightly slower than CME1, the two eruptions merge early on and appear to propagate away from the Sun as a single structure, possibly due to solar wind preconditioning caused by the prior CME (i.e., CME1), which is a phenomenon seen both in observations (e.g., Y. D. Liu et al., 2014, 2019; Temmer & Nitta, 2015) and in simulations (e.g., Desai et al., 2020; Scolini et al., 2020). The resulting CME‐driven shock and/or sheath is predicted to impact Venus (2012‐05‐01T19:35), Earth (2012‐05‐02T06:15), Mars (2012‐05‐05T23:33), and Saturn (2012‐06‐10T14:10). The merged ejecta is predicted to impact Venus (2012‐05‐02T00:42), Earth (2012‐05‐02T13:53), Mars (2012‐05‐06T06:31), and Saturn (2012‐06‐11T14:26).

## 5 In‐Situ Measurements

In this section, we present and analyze in‐situ data from Venus at 0.7 AU (Section 5.1), Earth at 1.0 AU (Section 5.2), and finally Saturn at 9.7 AU (Section 5.3). We remark that, although both propagation models shown in Section 4 estimated an impact at Mars, the main focus of this work is the study of the magnetic structure of the CMEs from eruption through heliospheric propagation. Since there was no spacecraft equipped with a solar wind‐sampling magnetometer in orbit around Mars at the time of the events presented here, this location is not included in our investigation.

### 5.1 Measurements at Venus

**Figure 7:**
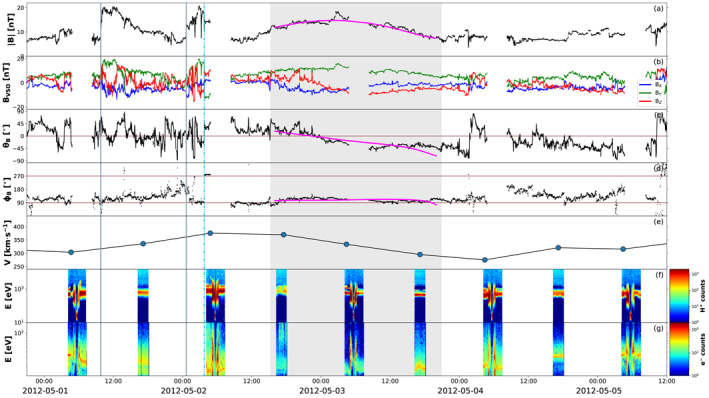
Measurements at Venus around the expected arrival time of the merged coronal mass ejection (CME1) and CME2. The parameters shown are: (a) magnetic field magnitude, (b) magnetic field components in Venus Solar Orbital (VSO) Cartesian coordinates, (c) θ and (d) ϕ angles of the magnetic field in VSO angular coordinates, (e) solar wind speed, and (f) proton and (g) electron energy distribution. The solid gray lines indicate two possible interplanetary shocks. The dash‐dotted blue line represents a possible start of the magnetic field rotation. The shaded green area marks the period characterized by flux rope signatures and enhanced magnetic field, corresponding to a magnetic ejecta. The magenta curves over magnetic field data within the ejecta show fitting results from applying the Elliptic–Cylindrical flux rope model of Nieves‐Chinchilla, Linton, et al. (2018).

The first impact location predicted by both models presented in Section 4 is Venus. On April 28, 2012, Venus was located ∼25° east of the Sun–Earth line, at 0.72 AU (see Figure 1). In‐situ measurements at Venus taken around the expected arrival time of CME1 and CME2 are shown in Figure 7, revealing the passage of a clear, albeit weak, interplanetary disturbance. In particular, two sudden increases in the magnetic field magnitude (at 2012‐05‐01T09:50 and 2012‐05‐02T00:39, marked by solid lines in Figure 7) may correspond to two interplanetary shocks, but it is not possible to establish this with certainty because of the lack of high‐cadence plasma data. Nevertheless, the solar wind speed displays an increase after each magnetic field jump, suggesting that the two structures may indeed coincide with shocks. These are followed by a period of enhanced magnetic field featuring a rotation in the $\theta_{B}$ component and a steady $\phi_{B}$ (shaded area in Figure 7), characteristic of a flux rope configuration. The exact boundaries (i.e., leading and trailing edge) of this magnetic ejecta are not straightforward to identify, in particular because the rotation seem to extend beyond the enhancement in the magnetic field magnitude (see also the dash‐dotted line in Figure 7, which appears to mark the beginning of a smoothly rotating region but is followed by a data gap). Hence, the identified ICME ejecta region (from 2012‐05‐02T15:14 to 2012‐05‐03T20:56) is based largely on the magnetic field magnitude (assumed to be greater than its surrounding material). Furthermore, it is unclear whether this structure corresponds to a single ejecta or to the merged CME1 and CME2, especially because of the lack of high‐cadence plasma measurements and a data gap in the middle of the flux rope. It has been shown, in fact, that the interaction of two CMEs may result in an ejecta that resembles a coherent, isolated magnetic flux rope (e.g., Kilpua et al., 2019; Lugaz & Farrugia, 2014).

Visual inspection of the magnetic field components within the ejecta reveals a (weak) rotation from north to south and a constant eastward direction, corresponding to a right‐handed, NES flux rope type. This was also the magnetic configuration of both CME1 and CME2 in the outer corona as inferred from the analysis of solar disc and coronagraph imagery (see Sections 3.1, 3.2). We also fit the structure using the Elliptic–Cylindrical (EC) analytical model of Nieves‐Chinchilla, Linton, et al. (2018), which is able to describe a magnetic flux rope topology with a distorted cross‐section, which may result from interactions with for example the solar wind. The results are shown over the magnetic field measurements in in Figure 7 and the full set of parameters obtained are reported in Table S1. Apart from vector magnetic field data, the model requires as input the CME average speed, for which we choose a value v=333 km$\cdot s^{-1}$ based on the three data points available. According to the EC model, the flux rope is right‐handed and has axis orientation (θ,ϕ) = (−10°,128°), fully consistent with a NES‐type structure. Furthermore, its cross‐section is rather distorted, with a distortion parameter δ=0.47 (δ is defined to be 1 for a circular cross‐section and 0 for maximum distortion).

### 5.2 Measurements at Earth

**Figure 8:**
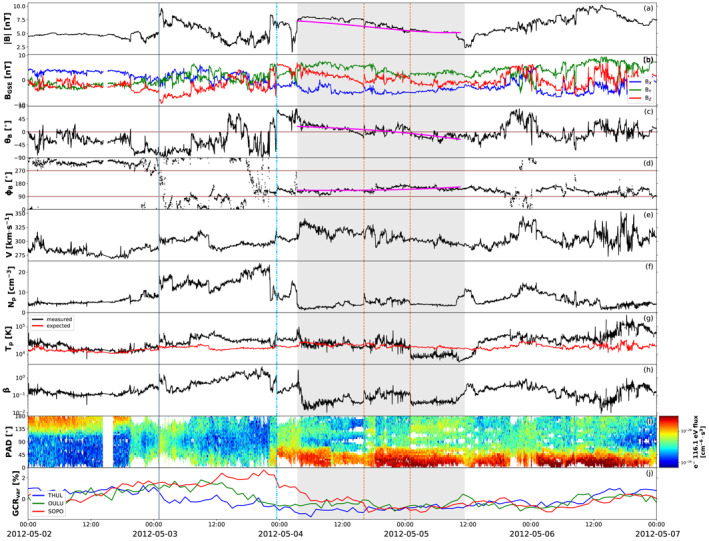
Measurements at Earth around the expected arrival time of the merged CME1 and CME2. The parameters shown are: (a) magnetic field magnitude, (b) magnetic field components in Geocentric Solar Ecliptic (GSE) Cartesian coordinates, (c) θ and (d) ϕ angles of the magnetic field in GSE angular coordinates, (e) solar wind speed, (f) proton density, (g) proton temperature (with the expected temperature defined by I. G. Richardson & Cane, 1995, shown in red), (h) plasma beta, (i) pitch angle distribution, and (j) galactic cosmic rays percentage variation (measured by three different neutron monitors on ground). The solid gray line marks the arrival of the interplanetary shock. The dash‐dotted blue line represents the beginning of the magnetic field rotation. The shaded gray area marks the magnetic cloud, with an interaction region delimited by dashed orange lines. The magenta curves over magnetic field data within the ejecta show fitting results from applying the Elliptic–Cylindrical flux rope model of Nieves‐Chinchilla, Linton, et al. (2018).

The next impact location predicted by the models presented in Section 4 is Earth, situated at 1.01 AU on April 28, 2012 (see Figure 1). In‐situ measurements at Earth taken around the expected arrival time of CME1 and CME2 are shown in Figure 8. The sequence of events starts with an interplanetary shock (at 2012‐05‐03T01:01, marked by a solid vertical line in Figure 8), followed by a decreasing magnetic field profile similarly to the first structure encountered at Venus (cf. Figure 7). We note that the shock is remarkably slow (v∼300 km$\cdot s^{-1}$), which has however been shown to be possible for slow CMEs characterized by significant expansion that travel through a slow upstream solar wind with a low magnetosonic speed (Lugaz, Farrugia, et al., 2017). We do not find signatures of a second shock at Earth. A second sharp increase in the magnetic field magnitude (similar to the one detected at Venus) is not associated with a solar wind speed jump and, furthermore, density and temperature feature a decrease. This suggests that the second discontinuity at Venus was also not a shock or that the second shock dissipated between 0.7 and 1.0 AU. The structure (between 2012‐05‐04T03:25 and 2012‐05‐05T11:23) following the second sharp magnetic field increase displays clear signatures of a magnetic cloud (shaded region in Figure 8), albeit with a low magnetic field strength and several complex characteristics. In particular, the center of the identified flux rope (between the dashed orange lines in Figure 8) features a region characterized by less smooth magnetic field, an irregular speed profile, and enhanced density and plasma beta. Furthermore, the proton temperature is relatively high throughout the first portion of the ejecta and finally drops below expected levels only after the high‐beta region (the same trend is observed for alpha particles, not shown here). Unfortunately, heavy ion composition or charge state data from the Advanced Composition Explorer (ACE) spacecraft are not available because of a data gap. Nevertheless, these characteristics are consistent with the merging CMEs or multiple magnetic clouds scenario described by, for example, Lugaz and Farrugia (2014) and Y. M. Wang et al. (2003), in which the central portion represents the interaction region between the two original ejecta (in this case, CME1 and CME2). Finally, we note that the interplanetary disturbance is associated with a weak (∼2% variation) Forbush decrease, registered at all neutron monitors considered here but with different onset times, ranging from the arrival of the interplanetary shock to the beginning of the magnetic field rotation (dash‐dotted line in Figure 8).

The magnetic cloud boundaries identified here coincide with the ones defined in the NASA–Wind ICME list (Nieves‐Chinchilla, Vourlidas, et al., 2018). Visual inspection of the magnetic field components within the ejecta reveal a very similar configuration to the one encountered at Venus (see Section 5.1), that is characterized by a north–south rotation of the helical field and an eastward axial field, forming a NES‐type flux rope. This picture is consistent with CME1 and CME2 (both of NES type in the outer corona, see Section 3.2) reconnecting in interplanetary space before reaching Venus and coalescing into a single NES flux rope. Again, we fit the structure using the EC model and assuming a CME average speed of v=310 km$\cdot s^{-1}$ directly from in‐situ data. The results are shown over the magnetic field measurements in Figure 8 and the full set of parameters obtained are reported in Table S1. The resulting flux rope is right‐handed, has axis orientation (θ,ϕ) = (2°,145°), and its distortion parameter is δ=0.46. We note that these results are compatible with those at Venus, that is consistent with a NES flux rope that is rather distorted.

### 5.3 Measurements at Saturn

**Figure 9:**
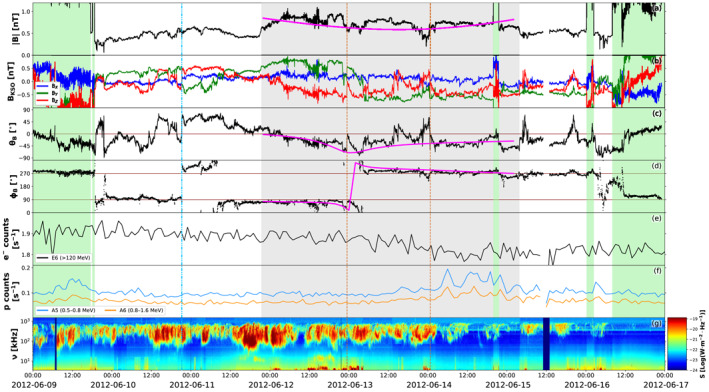
Measurements at Saturn around the expected arrival time of the merged CME1 and CME2. The parameters shown are: (a) magnetic field magnitude, (b) magnetic field components in Kronocentric Solar Orbital (KSO) Cartesian coordinates, (c) θ and (d) ϕ angles of the magnetic field in KSO angular coordinates, (e) electron (including penetrating GCR protons) and (f) proton count rates (with the corresponding LEMMS channels and proton energy ranges indicated), and (g) dynamic spectrum of SKR spectral flux density normalized to 1 AU. Green‐shaded regions correspond to periods in which Cassini was inside the Kronian magnetosheath. The beginning of the period featuring magnetic field rotation is marked with the dash‐dotted blue line, and the period of enhanced magnetic field magnitude is shaded in gray, with a possible interaction region indicated by the dashed orange lines. The magenta curves over magnetic field data within the ejecta show fitting results from the Elliptic–Cylindrical flux rope model of Nieves‐Chinchilla, Linton, et al. (2018).

Finally, the last location where an arrival of CME1 and CME2 is predicted is Saturn, which was positioned at 9.73 AU from the Sun and ∼12° east of Earth on April 28, 2012 (see Figure 1). Even though CMEs usually take a relatively long time to reach ∼10 AU (about a month; e.g., Prangé et al., 2004), Saturn's 29‐year orbit results in the planet moving about 1° in longitude per month and, thus, can be considered essentially fixed in space throughout the analyzed period. In‐situ measurements at Saturn taken around the expected arrival time of CME1 and CME2 are shown in Figure 9. Providentially, Cassini exited the Kronian magnetosheath between June 9–16, 2012 and was fully immersed in the solar wind during most of this time interval. On the other hand, Cassini's plasma spectrometer was permanently turned off on June 2, 2012 (i.e., just a week before the start of the data shown in Figure 9) due to short circuits in the instrument, hence there are no measurements such as solar wind speed and proton density available. Nevertheless, we complement magnetic field data with particle measurements and radio observations of Saturn's Kilometric Radiation (SKR; e.g., Kaiser et al., 1984; Lamy et al., 2008; Warwick et al., 1981). The SKR is Saturn's primary radio emission and is generated by auroral electrons that are accelerated along field lines rooted around Saturn's auroral oval (e.g., Lamy, 2017; Lamy et al., 2009). The emission is highly dependent on solar wind conditions (e.g., Bradley et al., 2020; Clarke et al., 2009; Desch, 1982; Kurth et al., 2005; Lamy et al., 2018), and previous studies have reported SKR enhancements concurrently with the passage of ICMEs (e.g., Crary et al., 2005; Palmaerts et al., 2018) and SIRs (e.g., Badman et al., 2008; Kurth et al., 2016).

First of all, the magnetic field measurements shown in Figure 9 reveal the passage of a (weak) disturbance, since the (ambient) interplanetary magnetic field around Saturn's orbit has usual magnitudes below 0.5 nT (e.g., Jackman et al., 2008; Echer, 2019). In this case, the magnetic field magnitude reaches values just under 1 nT, which is rather low (cf. the ∼2 nT measurements reported by Witasse et al., 2017, for an ICME in 2014 November), but is on the other hand consistent with the “weakness” of the transient measured at Earth (∼7.5 nT, see Figure 8). We do not find signatures of an interplanetary shock, but we could identify a period of smoothly rotating magnetic field (starting at 2012‐06‐10T21:24, marked by the dash‐dotted blue line in Figure 9) that includes the interval of enhanced magnetic field magnitude (between 2012‐06‐11T21:24 and 2012‐06‐15T03:50, shaded gray region in Figure 9). We note that the central portion of this interval (bounded between the dashed orange lines in Figure 9) is characterized by a more irregular $\theta_{B}$ component that is reminiscent of the one encountered at Earth (see Figure 8), that is indicating a possible interaction region. In order to confirm that an interplanetary disturbance has indeed impacted Saturn, we also examine energetic particle observations. In this regard, Roussos et al. (2018, 2020) showed that Cassini observations of solar energetic particle (SEP) and GCR transients can be used to identify disturbed solar wind conditions around Saturn, especially due to CMEs and SIRs. This is because SEPs and GCRs are able to penetrate Saturn's magnetosphere, hence they can be monitored at all times (e.g., Roussos et al., 2008, 2011). During our period of interest, we find signatures of a Forbush decrease in the GCR data that are indirectly monitored with the LEMMS electron channel E6 (due to penetrating GCR protons; Roussos et al., 2019). This event was included by Roussos et al. (2018) in their list of SEP and GCR transients at Saturn between 2004 and 2016. The decrease commenced around 10 June 2012 and reached its minimum around 14 June, concurrently with an increase in the LEMMS proton channels A5/A6 (the recovery phase of the Forbush decrease follows the plotted interval and is shown in its full extent by Roussos et al., 2018). Furthermore, SKR data feature increased emission between 2012‐06‐10T13:08 and 2012‐06‐14T02:16, that is over an interval that is in agreement with the Forbush decrease. The temporal extent of the SKR intensification is consistent with enhancements triggered by solar wind pressure fronts, as opposed to transient ones triggered by planetary rotation (lasting a few hours; e.g., Reed et al., 2018). Finally, we note a peak in proton counts occurring on June 9, 2012 shortly before the interval of enhanced SKR emission. It is unclear whether this structure has a magnetospheric or solar origin, especially since it was observed when Cassini was inside the Kronian magnetosheath. Nevertheless, the features detected during June 10–14, 2012 (in magnetic field, particles, and SKR) occur approximately at the same time, and strongly suggest that a solar transient, and in particular an ICME, impacted Saturn during the observed period.

The magnetic configuration of the entire structure characterized by smoother magnetic field components (i.e., from the dash‐dotted blue line to the end of the shaded gray area in Figure 9) exhibits a larger rotation than that expected for an axial‐symmetric flux rope (the $\phi_{B}$ component rotates as west–east–west, resulting in a 360° rotation in the $B_{Y}$–$B_{Z}$ hodogram). Similar events displaying a rotation greater than 180° in the magnetic field were analyzed at 1 AU by Nieves‐Chinchilla et al. (2019), who suggested that such events can be interpreted as flux ropes with significant curvature and/or distortion or as more complex topologies, such as a spheromak or a double flux rope. Since it is not unusual for interplanetary transients to interact and possibly merge by 1 AU, one may expect even more interaction and complexity in structures detected in the outer heliosphere (e.g., Hanlon et al., 2004; Prise et al., 2015). Hence, it is not possible to establish with certainty whether the observed configuration stems from a single, intrinsically complex structure, from the interaction of different structures, or if the region preceding the period of enhanced magnetic field magnitude corresponds to a “smoothed” sheath. Nevertheless, considering only the shaded gray region in Figure 9, visual inspection of the magnetic field yields an ESW flux rope type, still right‐handed but corresponding to a ∼90° counterclockwise rotation of the configuration found at Venus and Earth (NES). Again, we fit the structure using the EC model. In this case, due to the lack of plasma data at Saturn, we assume an average CME speed of v=380 km$\cdot s^{-1}$ by considering the transit times of the flux rope leading and trailing edges from Earth to Saturn. Fitting results are shown over the magnetic field measurements in in Figure 9 and the full set of parameters obtained are reported in Table S1. The resulting structure is right‐handed (as expected), but its axis has orientation (θ,ϕ) = (−9°,3°), that is consistent with a low‐inclination flux rope. Although the reconstructed magnetic field components fit well to the data, the observed east–west rotation is attributed in the model to a crossing more parallel to the central axis of a low‐inclination flux rope, rather than a crossing perpendicular to the axis of a high‐inclination one. Furthermore, the resulting distortion parameter is δ=0.21, indicating that the structure is highly distorted.

## 6 Discussion

In this section, we synthesize the multi‐spacecraft observations, modeling results, and interpretations presented in Sections 3, 4, and 5 and discuss them in the context of two main aspects: the propagation of the interacting CME1 and CME2 to 10 AU (Section 6.1) and the evolution of their magnetic structure from the Sun to Saturn (Section 6.2).

### 6.1 CME Propagation

CME1 and CME2 erupted ∼5 h apart from the southeastern quadrant of the Earth‐facing disc, and their source regions were separated by ∼30° in latitude (see Section 3.1). Through the solar corona (see Section 3.2), CME1 could be seen to deflect significantly toward the solar equator (its apex position changed from approximately S45E15 to S12E18), whilst CME2 propagated radially (its apex direction could be considered basically unchanged, from S15E20 at the Sun to S12E24 in the corona). This resulted in the two CMEs traveling away from the Sun in close succession and on a very similar trajectory. Despite CME2 appearing slightly slower (by ∼100 km$\cdot s^{-1}$) than CME1 at an altitude of ∼15 $R_{⊙}$, the two eruptions could not be clearly distinguished in HI imagery (see Section 3.3), possibly indicating that they interacted somewhere in the HI1 field of view. This outcome occurs also in the Enlil simulation (Section 4.2), where the CMEs appear as a merged structure throughout the heliospheric domain even if they were inserted separately at the inner boundary of 21.5 $R_{⊙}$. We suggested that this scenario can be attributed to solar wind preconditioning (e.g., Temmer et al., 2017), which allowed CME2 to travel through a rarefied background experiencing little to no drag and thus to run into CME1. As a consequence, we considered CME1 and CME2 as a single, interacting structure when estimating their propagation throughout the heliosphere.

In Section 4, we used two models to evaluate the arrival times of the interacting CME1 and CME2 at different locations and to aid interpretation of the in‐situ measurements shown in Section 5. The two propagation models that we employed are substantially different in their physics, assumptions, and observational input: the SSSE model (Section 4.1) consists of a 2D circular cross‐section reconstructed using HI data and is then propagated outwards assuming constant acceleration, whilst Enlil (Section 4.2) is a full 3D MHD model of the heliosphere in which we inserted CME1 and CME2 as hydrodynamic pulses and with input parameters based on coronagraph imagery. Nevertheless, both techniques estimated impacts at the same locations (Venus, Earth, Mars, and Saturn) and within reasonable temporal windows compared to the actual in‐situ observations. In particular, the predicted arrival time at Saturn was remarkably accurate (with same‐day precision) in the case of Enlil, and about 3 days off for SSSE (corresponding to a 7% error for a ∼43‐day propagation). We note that, regardless of these encouraging results, both models have made a number of simplifying assumptions. For example, the treatment of CMEs as hydrodynamic pulses within Enlil is likely to affect the predicted arrival times due to the absence of internal magnetic forces that can contribute to the acceleration profile. However, as a CME travels away from the Sun these forces are expected to be less influential, especially at radial distances of a few AU and through the outer heliosphere. The SSSE model, on the other hand, treats CMEs as self‐similarly propagating spherical fronts under the assumption of constant half‐width and acceleration, from which it would be unrealistic to expect precise estimates at 10 AU. Nevertheless, these results suggest that even simplifying models can be used to successfully approximate a CME arrival time window up to the outer heliosphere.

**Figure 10:**
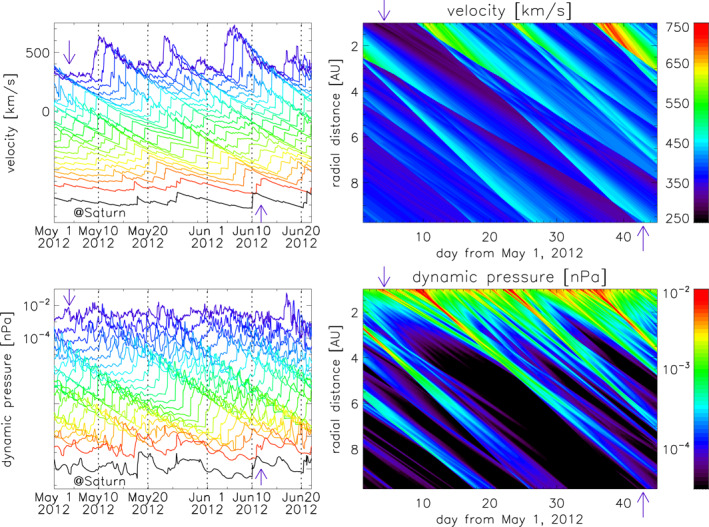
Solar wind propagated from 1 to 9.75 AU using the Tao et al. (2005) model. The top panels show solar wind speed, whilst the bottom panels show dynamic pressure. Results are shown in both line (left) and contour (right) formats. In the line plots, the values for speed and dynamic pressure beyond 1 AU are progressively shifted toward lower values for visibility (values at different heliocentric distances can be read in the contour plots). In the line plots, the blue lines show measurements at Earth, the red lines show solar wind propagated to 9.75 AU along the Sun–Earth line, and the black lines at the bottom show model results shifted to Saturn's position. In all panels, the downward‐pointing arrows mark the ICME ejecta arrival time at Earth (see Figure 8), whilst the upward‐pointing arrows mark the ICME ejecta arrival time at Saturn (see Figure 9).

As a further indication of the solar wind propagation that occurred between 1 and 10 AU, we employ the 1D numerical MHD model of Tao et al. (2005), which uses in‐situ measurements from Earth or the STEREO spacecraft to estimate the interplanetary conditions further out in the heliosphere, such as the orbits of Jupiter (e.g., Dunn et al., 2020), Saturn (e.g., Provan et al., 2015), and Uranus (e.g., Lamy et al., 2017). In this case, we use data at Earth (closest to Saturn in longitude, see Figure 1) and propagate the measurements up to ∼10 AU along the Sun–Earth line. The results for speed and dynamic pressure are shown in Figure 10. The ICME at Earth was followed by a high‐speed stream (HSS), which is estimated to have arrived at Saturn around June 10, 2012. The solar wind preceding this HSS (which includes the ICME measurements shown in Figure 8) is expected to be caught up by the fast stream around May 25, 2012 at a heliocentric distance of ∼5 AU, possibly resulting in compression and acceleration of the ICME from behind (this is consistent with the Enlil simulation, see Figure 6b). The arrival time of these features at 10 AU matches quite well with the Enlil results (see Section 4.2) and the in‐situ measurements at Saturn (see Section 5.3), further confirming the likelihood of our connection.

We also explored the level of interaction and possible merging between CME1 and CME2 through interplanetary space. We emphasise that, whilst the Enlil simulation estimated the two eruptions to merge well before reaching Mercury's orbit, it is not possible to fully model the nature and outcome of their interaction due to the lack of an internal magnetic field in the purely hydrodynamic representation of CMEs. Hence, our interpretation is entirely based on the in‐situ observations presented in Section 5. At Venus, we did not find clear indications of interaction, possibly because of the lack of high‐cadence plasma data and a data gap exactly at the center of the flux rope interval that we identified (see Figure 7). At Earth, a seemingly single magnetic cloud structure displayed less smooth magnetic field, variable speed, and enhanced plasma parameters at its center, which could be considered signatures of the interaction between CME1 and CME2 (see Figure 8). At Saturn, the region that we selected as the magnetic ejecta featured similar magnetic field characteristics at its center as the ones encountered at Earth (see Figure 9). However, we could not determine with confidence whether the observed structure corresponded entirely to the ejecta detected at Venus and Earth, or whether additional material was added to it via further interaction with nearby structures in the solar wind. At all locations, fitting the entire ejecta interval as a single flux rope yielded the best results, suggesting that, if interaction was present, it resulted in the two eruptions slowly merging and traveling as a single magnetic structure, rather than in more extreme outcomes such as the second CME overcoming and compressing the first. This is plausible, considering that CME1 and CME2 were ejected close in time and with comparable speeds. An alternative interpretation is that CME1 skimmed the inner planets and its signatures were observed following the first interplanetary shock, and the entire ejecta intervals at Venus and Earth belonged on the other hand to CME2.

### 6.2 CME Magnetic Structure

Both CME1 and CME2 erupted from the Sun as right‐handed flux ropes, but with slightly different orientations according to our analysis of solar disc imagery (Section 3.1): CME1 featured a low‐inclination NES type, whilst CME2 displayed a high‐to‐intermediate inclination between an ESW and a NES type. In the solar corona, CME1 was observed to largely maintain its orientation, whilst CME2 appeared to have slightly rotated in a clockwise direction, from which we inferred that both eruptions consisted of NES‐type flux ropes based on coronagraph imagery and reconstructions (Section 3.2). The two CMEs were hard to distinguish as separate structures in HI data (Section 3.3), suggesting that they interacted in some capacity in the inner heliosphere. At Venus (Section 5.1), we found a single flux rope signature of NES type (based on both visual inspection and flux rope fitting), which did not display evident indications of interaction (possibly because of lacking high‐cadence plasma data and magnetic field measurements at the center of the structure). Nevertheless, the whole interplanetary distrubance (including shock and sheath material) was rather long in duration (∼2.5 days), suggesting that it may be linked to more than one parent eruption. At Earth (Section 5.2), we again found a long‐duration (∼2.5 days) disturbance culminating in a seemingly single magnetic cloud of NES type (based on both visual inspection and flux rope fitting). However, we observed characteristic interaction signatures at the center of the flux rope, including enhanced plasma density, temperature, and beta, as well as an irregular speed profile and a less smooth magnetic field in the $\theta_{B}$ direction. Finally, at Saturn (Section 5.3), we found an extended (∼4 days in duration) disturbance mostly characterized by smoothly rotating field. We associated a period of enhanced magnetic field magnitude with a magnetic ejecta, noting that it featured at its center an irregular magnetic field profile (especially in $\theta_{B}$) reminiscent of the one encountered at Earth and attributed to interaction signatures. Its corresponding flux rope type was determined to be ESW based on visual inspection, whilst flux rope fitting yielded a low‐inclination structure with its axis pointing back toward the Sun. The chirality was found to be consistently right‐handed at all in‐situ locations.

The overall picture extrapolated from the set of observations described above is that CME1 and CME2 started to interact in the inner heliosphere (roughly in the HI1 field of view and before the orbit of Venus). The distinct NES ejecta measured at Venus and Earth suggests that reconnection between the trailing edge of CME1 and the leading edge of CME2 resulted in a single flux rope that maintained the original orientation of its constituent parts. The resulting magnetic structure of interacting CMEs has been analyzed via simulations by, for example, Lugaz et al. (2013) and Schmidt and Cargill (2004). Two flux ropes with the same twist and orientation (in this case, NES) are favorably configured for magnetic reconnection during interaction, at least at the interface between the two, which is where antiparallel fields meet. These conclusions were also found by Kilpua et al. (2019), who analyzed two interacting CMEs in June 2012 that left the solar corona as NES types and were observed at Venus at the beginning of their interaction and at Earth as a single NES flux rope. The structure observed at Saturn, if corresponding in its entirely to the merged flux ropes detected in the inner heliosphere, would be consistent either with a modest (<90°) counterclockwise rotation in the north–south direction (based on the ESW configuration retrieved from visual inspection) or with a ∼130° rotation along the equatorial plane (based on flux rope fitting) between 1 and 10 AU, possibly as a result of the pressure exerted by a following HSS (see also the discussion in Section 6.1).

**Figure 11:**
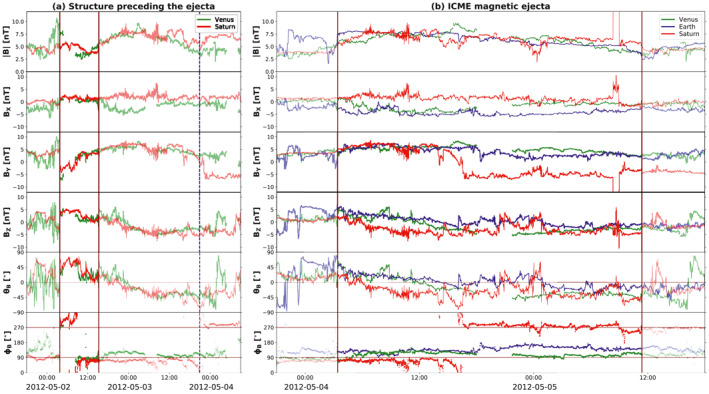
Magnetic field mapping between (a) Venus and Saturn for the small structure preceding the ejecta, and (b) Venus, Earth, and Saturn for the full ICME ejecta, using the method in Good et al. (2018). In (a) measurements at Saturn have been temporally shifted and scaled to match those at Venus. The dashed line marks the identified ejecta trailing edge at Venus. In (b), measurements at Venus and Saturn have been temporally shifted and their magnitudes have been scaled to match data taken near Earth.

Finally, in order to investigate how the observed structure at Saturn relates to the ones at Venus and Earth, we employ the magnetic field mapping technique of Good et al. (2018), used to determine the arrival time and magnetic field of different plasma parcels in an ejecta between radially separated spacecraft. The technique takes into account expansion as a CME travels through interplanetary space, and assumes that each ejecta features a monotonically increasing/decreasing speed profile determined by its leading and trailing edge speeds (calculated on the basis of their arrival times from one location to the next). Magnetic field mapping results for two different structures are shown in Figure 11. The full identified magnetic ejecta at Venus, Earth, and Saturn (i.e., the shaded gray areas in Figures 7, 8, 9) is mapped in panel (b), whilst in panel (a) the smaller magnetic structure preceding the ejecta (i.e., from the dash‐dotted blue lines to the ejecta leading edges in Figures 7 and 9) is mapped between Venus and Saturn. In both panels, the measurements bounded by solid vertical lines are mapped according to the Good et al. (2018) technique, whilst the preceding and following data are propagated at the resulting leading and trailing edge speeds, respectively. In Figure 11a, it is evident that, despite a data gap at Venus, the magnetic field mapping shows good agreement between the two spacecraft in all the magnetic field components. We also note that, by propagating the following measurements at Saturn at the resulting trailing edge speed (387 km$\cdot s^{-1}$), good agreement between the two data sets continues to be displayed roughly until the ejecta trailing edge at Venus and the start of the identified interaction region at Saturn (i.e., close to the first dashed orange line in Figure 9). This would suggest that the original ejecta resulting from the interaction of CME1 and CME2 may be linked to approximately the first third of the flux rope interval identified at Saturn, and that the full ejecta may on the other hand correspond to a MIR, which as mentioned in the Introduction is a dominating structure in the outer heliosphere. Contrarily, the magnetic field mapping in Figure 11b shows good agreement in all the magnetic field components between Venus and Earth, but a substantial difference in the $\phi_{B}$ component at Saturn in the second half of the ejecta (which rotates from east to west). This is however not surprising, since the structure at Saturn was found to display a different orientation from that at Venus and Earth. Furthermore, the central, more turbulent portion of magnetic field measurements found at Earth and Saturn appears to match relatively well. According to these results, it is not possible to establish with certainty the relationship between measurements at Saturn and those at Venus and Earth (i.e., whether the original flux rope corresponds to the full ejecta interval at Saturn or to a portion of it). We emphasise that the magnetic field mapping technique of Good et al. (2018) assumes monotonically increasing/decreasing speed, which is not a realistic assumption as far from the Sun as 10 AU, where it is likely for a CME to have accelerated/decelerated multiple times via continued interactions with the ambient solar wind (evident also in the high distortion parameter found at Saturn). Nevertheless, this method appears to work well at least for a qualitative comparison of structures observed in the inner and outer heliosphere.

## 7 Conclusions

In this work, we have analyzed the eruption and evolution of two CMEs (CME1 and CME2) that left the Sun on April 28, 2012 just a few hours apart. After observing their early evolution through the solar corona, we found indications of interaction in HI imagery, and we eventually identified signatures of a single ejecta at the in‐situ locations that we considered (Venus, Earth, and Saturn), suggesting that the two eruptions had merged. This study represents the first detailed analysis of the magnetic structure of CME flux ropes from the Sun to 10 AU, taking advantage of remote‐sensing observations of the Sun, its corona, and interplanetary space, as well as in‐situ measurements at three planets. Whilst the chirality of CME1 and CME2 at the Sun and of the flux rope ejecta detected in situ was found to be consistently right‐handed, the flux rope type presented some changes. At the Sun, CME1 erupted as a NES flux rope and CME2 displayed a high‐to‐intermediate inclination (between ESW and NES). At Venus and Earth, the single observed ejecta was of type NES, whilst at Saturn we found a structure featuring an ESW rotation that is consistent with a rotation on either the meridional or the equatorial plane. We could not determine with certainty whether the ejecta that we identified at Saturn consisted entirely of material from CME1 and CME2, or whether additional material was gathered on the way to 10 AU, thus forming a MIR.

The CME propagation models that we used to estimate the impact locations of CME1 and CME2 (which were treated as a single, merged structure) across the heliosphere provided useful insights necessary to interpret the in‐situ observations, and were reasonably well‐timed compared to the actual measurements. However, in‐depth understanding of how CME magnetic fields evolve throughout interplanetary space and interact with other CMEs or the ambient solar wind still remains an arduous task. In the case of the events under study, an “intermediate” observer between Earth and Saturn, perhaps around 5 AU, may have helped shed more light on the evolution of solar transients beyond 1 AU, especially as it may have been possible to catch the ICME ejecta at the beginning of its interaction with the following HSS. Nevertheless, this study remarks the importance of multi‐point studies of CMEs and the advantages to be gained by analyzing data from both heliospheric and planetary missions, which are necessary steps to undertake in order to deepen our current understanding of the structure and evolution of solar transients in the outer heliosphere.

## Supporting information

Supporting Information S1

Movie S1

Movie S2

Movie S3

## Data Availability

The HELCATS catalogues are available at https://www.helcats-fp7.eu. Images and additional information on the 28 April 2012 CME(s) are available at https://www.helcats-fp7.eu/catalogues/event_page.html?id=HCME_A__20120428_01 (STEREO‐A viewpoint) and https://www.helcats-fp7.eu/catalogues/event_page.html?id=HCME_B__20120428_02 (STEREO‐B viewpoint). Enlil simulation results have been provided by the Community Coordinated Modeling Center (CCMC) at NASA Goddard Space Flight Center through their public Runs on Request system (http://ccmc.gsfc.nasa.gov). The full Enlil simulation results are available at https://ccmc.gsfc.nasa.gov/database_SH/Erika_Palmerio_031021_SH_3.php (run id: Erika_Palmerio_031021_SH_3). The NASA–Wind ICME list can be found at https://wind.nasa.gov/ICMEindex.php. Solar disc and coronagraph data from SDO, SOHO, and STEREO are openly available at the Virtual Solar Observatory (VSO; https://sdac.virtualsolar.org/). These data were processed and analysed trough SunPy (SunPy Community et al., 2015, 2020), IDL SolarSoft (Freeland & Handy, 1998), and the ESA JHelioviewer software (Müller et al., 2017). Level‐2 processed STEREO/HI data were obtained from the UK Solar System Data Centre (UKSSDC; https://www.ukssdc.ac.uk/solar/stereo/data.html). VEX data are openly available at ESA's Planetary Science Archive (PSA; Besse et al., 2018), accessible at https://archives.esac.esa.int/psa. These data were processed and analysed with the aid of the irfpy library (https://irfpy.irf.se/irfpy/index.html). Wind data are publicly available at NASA's Coordinated Data Analysis Web (CDAWeb) database (https://cdaweb.sci.gsfc.nasa.gov/index.html/). NMDB data are publicly available at http://www.nmdb.eu. Cassini data are openly available at the Planetary Plasma Interactions (PPI) Node of NASA's Planetary Data System (PDS), accessible at https://pds-ppi.igpp.ucla.edu. All in‐situ spacecraft data can also be found on the Automated Multi‐Dataset Analysis (AMDA; Génot et al., 2021) tool at the address http://amda.irap.omp.eu. Solar wind data propagated with the Tao et al. (2005) model can be browsed at the AMDA website and at the HelioPropa tool, accessible at http://heliopropa.irap.omp.eu.
