# Supplementary material for: Magnetic Structure and Propagation of Two Interacting CMEs From the Sun to Saturn
Source: J Geophys Res Space Phys. 2021 Nov 3;126(11):e2021JA029770. doi: 10.1029/2021JA029770 (PMC9286593; doi:10.1029/2021JA029770)
Supplement: Supplementary file 1 — Supporting Information S1 [file JGRA-126-0-s002.pdf]

Supporting Information for "Magnetic Structure and Propagation of Two Interacting CMEs from the Sun to Saturn"

Erika Palmerio[1,2], Teresa Nieves-Chinchilla[3], Emilia K. J. Kilpua[4], David Barnes[5], Andrei N. Zhukov[6,7], Lan K. Jian[3], Olivier Witasse[8], Gabrielle Provan[9], Chihiro Tao[10], Laurent Lamy[11,12], Thomas J. Bradley[9], M. Leila Mays[3], Christian Möstl[13,14], Elias Roussos[15], Yoshifumi Futaana[16], Adam Masters[17], and Beatriz Sánchez-Cano[9]

[1]Space Sciences Laboratory, University of California–Berkeley, Berkeley, CA, USA
[2]CPAESS, University Corporation for Atmospheric Research, Boulder, CO, USA
[3]Heliophysics Science Division, NASA Goddard Space Flight Center, Greenbelt, MD, USA
[4]Department of Physics, University of Helsinki, Helsinki, Finland
[5]STFC RAL Space, Rutherford Appleton Laboratory, Harwell Campus, Oxfordshire, UK
[6]Solar–Terrestrial Centre of Excellence—SIDC, Royal Observatory of Belgium, Brussels, Belgium
[7]Skobeltsyn Institute of Nuclear Physics, Moscow State University, Moscow, Russia
[8]ESTEC, European Space Agency, Noordwijk, Netherlands
[9]School of Physics and Astronomy, University of Leicester, Leicester, UK
[10]Institute of Space and Astronautical Science, Japan Aerospace Exploration Agency, Japan
[11]LESIA, Observatoire de Paris, PSL, CNRS, UPMC, Université Paris Diderot, Meudon, France
[12]LAM, Pythéas, Aix Marseille Université, CNRS, CNES, Marseille, France
[13]Space Research Institute, Austrian Academy of Sciences, Graz, Austria
[14]Institute of Geodesy, Graz University of Technology, Graz, Austria
[15]Max Planck Institute for Solar System Research, Göttingen, Germany
[16]Swedish Institute of Space Physics, Kiruna, Sweden
[17]The Blackett Laboratory, Imperial College London, London, UK

Contents of this file

1. Table S1
2. Captions for Movies S1 to S3

Corresponding author: Erika Palmerio, `epalmerio@berkeley.edu`

Table S1

Table 1. Fitting results from applying the Ellyptic–Cylindrical (EC) model to the flux rope intervals identified at Venus, Earth, and Saturn (grey shaded areas in Figures 7, 8, and 9 in the main manuscript). The input parameters for each fit are vector magnetic field data and average speed of the flux rope. The values for speed that we use are 333 km·s^{-1} at Venus, 310 km·s^{-1} at Earth, and 380 km·s^{-1} at Saturn.

Location	$C1$	B_0^y [nT]	δ	θ [°]	ϕ [°]	ξ [°]	y_0 [AU]	R [AU]	H	ρ	χ^2
Venus	1.74	33.3	0.47	−9.8	127.7	89.1	−0.05	0.13	+	0.81	0.22
Earth	1.99	16.0	0.46	2.0	145.4	42.1	−0.03	0.12	+	0.76	0.24
Saturn	0.15	1.4	0.21	−9.0	3.0	23.9	0.01	0.08	+	0.62	0.33

Note. $C1$: ratio of the azimuthal to axial current at the outer boundary of the flux rope; B_0^y: axial magnetic field; δ: distortion parameter ($\delta = 1$ for a circular cross-section and $\delta = 0$ for maximum distortion); θ: axis latitude; ϕ: axis longitude; y_0: closest approach to the flux rope axis; ξ: propagation angle (i.e., rotation around the flux rope axis) ; R: flux rope radius; H: helicity sign (or chirality); ρ: correlation coefficient; χ^2: chi-squared value.

Caption for Movie S1

Eruption of CME0, CME1, and CME2 on 28 April 2012 as seen from (left) STEREO/SECCHI/EUVI-A, (middle) SDO/AIA, and (right) STEREO/SECCHI/EUVI-B in the 193/195 Å (top) and 304 Å (bottom) channels.

Caption for Movie S2

Propagation through the solar corona of CME0, CME1, and CME2 as seen in running-difference images from (a) STEREO/SECCHI/COR2-A, (b) SOHO/LASCO/C2, and (c) STEREO/SECCHI/COR2-B.

Caption for Movie S3

Propagation through the inner heliosphere of CME1 and CME2 as seen in running-difference images from the (a) HI1-A and (b) HI1-B cameras onboard the STEREO spacecraft.
